# Marshalin, a microtubule minus-end binding protein, regulates cytoskeletal structure in the organ of Corti

**DOI:** 10.1242/bio.20135603

**Published:** 2013-09-17

**Authors:** Jing Zheng, David Furness, Chongwen Duan, Katharine K. Miller, Roxanne M. Edge, Jessie Chen, Kazuaki Homma, Carole M. Hackney, Peter Dallos, Mary Ann Cheatham

**Affiliations:** 1 Department of Otolaryngology – Head and Neck Surgery, Feinberg School of Medicine, Northwestern University, Chicago, IL 60611, USA; 2 Hugh Knowles Center for Clinical and Basic Science in Hearing and Its Disorders, Northwestern University, Evanston, IL 60208, USA; 3 School of Life Sciences, Institute for Science and Technology in Medicine (ISTM), Keele University, Keele ST5 5BG, UK; 4 Center for Molecular Neurobiology (ZMNH), University Medical Center Hamburg-Eppendorf, D-20251 Hamburg, Germany; 5 Department of Communication Sciences and Disorders, Northwestern University, Evanston, IL 60208, USA; 6 Department of Biomedical Science, The University of Sheffield, Western Bank, Sheffield S10 2TN, UK

**Keywords:** Microtubule minus-end binding protein, Noncentrosomal MTOC, Cochlea, CAMSAP3, Nezha, Patronin

## Abstract

Dramatic structural changes in microtubules (MT) and the assembly of complicated intercellular connections are seen during the development of the cellular matrix of the sense organ for hearing, the organ of Corti. This report examines the expression of marshalin, a minus-end binding protein, during this process of cochlear development. We discovered that marshalin is abundantly expressed in both sensory hair cells and supporting cells. In the adult, prominent marshalin expression is observed in the cuticular plates of hair cells and in the noncentrosomal MT organization centers (MTOC) of Deiters' and pillar cells. Based upon differences in marshalin expression patterns seen in the organ of Corti, we identified eight isoforms ranging from 863 to 1280 amino acids. mRNAs/proteins associated with marshalin's isoforms are detected at different times during development. These isoforms carry various protein–protein interacting domains, including coiled-coil (CC), calponin homology (CH), proline-rich (PR), and MT-binding domains, referred to as CKK. We, therefore, examined membranous organelles and structural changes in the cytoskeleton induced by expressing two of these marshalin isoforms *in vitro*. Long forms containing CC and PR domains induce thick, spindle-shaped bundles, whereas short isoforms lacking CC and PR induce more slender variants that develop into densely woven networks. Together, these data suggest that marshalin is closely associated with noncentrosomal MTOCs, and may be involved in MT bundle formation in supporting cells. As a scaffolding protein with multiple isoforms, marshalin is capable of modifying cytoskeletal networks, and consequently organelle positioning, through interactions with various protein partners present in different cells.

## Introduction

**Fig. 1.**
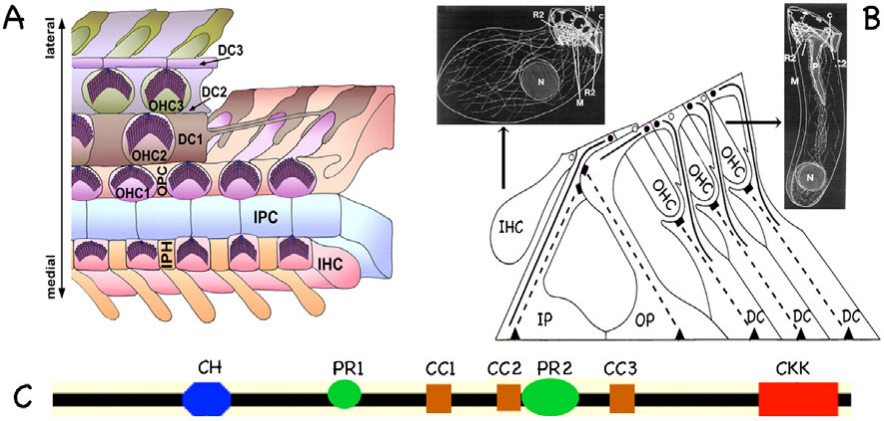
Anatomical details of the organ of Corti and its components. (A) Cartoon of the Organ of Corti (OC). Outer (OHC1–OHC3) and inner hair cells (IHC) are interdigitated with several types of distinct nonsensory supporting cells (SCs): the inner phalangeal cells (IPHs), inner pillar cells (IPCs), outer pillar cells (OPCs), and three rows of Deiters' cells (DC1–DC3s). (B) A cartoon depicting MTs in the OC. Centrosomal MT bundles: solid lines. Noncentrosomal MT bundles: broken lines. Noncentrosomal MTOCs near the basilar membrane: black triangles. Noncentrosomal MTOCs in the middle and upper regions of the sense organ: black rectangles. Circles represent centrosomes in SCs (filled circles) and HCs (open circles). Representations of MT networks in OHCs and IHCs are displayed in the upper right and left corners, respectively. (A and B are modified from Kelly and Chen, 2009; Steyger et al., 1989; Tucker et al., 1998). (C) Cartoon showing different domains of the Marshalin molecule. CKK: tubulin-binding domain (red). CH: calponin homology domain (blue). CC: coiled-coil domain (brown). PR: (proline-rich) region (green).

The organ of Corti, the sense organ of hearing in mammals, develops from relatively simple epithelial cells into a complex group of highly polarized hair cells (HCs) and their surrounding supporting cells (SCs) (Fig. 1A) (Kelly and Chen, 2009; Rida and Chen, 2009). Inner hair cells (IHCs) function as sensory receptors conveying sound-related information to the central nervous system, while outer hair cells (OHCs) amplify the mechanical signals delivered to IHCs (Dallos, 1992; Dallos et al., 2008). Supporting cells include, but are not limited to, Deiters' cells (DC), inner phalangeal cells (IPC), outer (OP) and inner pillar cells (IP). Pillar cells form the triangular tunnel of Corti with IHCs and OHCs positioned on either side. The apical portions of IHCs, OHCs, IPCs, IPs, OPs, and the phalangeal processes of DCs are connected through tight junctions to form the reticular lamina, which separates endolymph from perilymph. OHC basal portions are secured within Deiters' cups. Various intercellular structures, such as tight junctions, adhesive junctions, desmosomes, and gap junctions are found within the organ of Corti (Slepecky, 1996; Nunes et al., 2006). Even though both HCs and SCs are highly polarized, MT arrangements in these cell types differ (Fig. 1B). For example, sensory hair cells display loose microtubule (MT) networks with each MT containing 13 protofilaments (Slepecky and Chamberlain, 1985; Steyger et al., 1989; Furness et al., 1990; Slepecky and Ulfendahl, 1992). In contrast, supporting cells contain dense bundles made up of thousands of individual MT filaments, each composed of 15 protofilaments (Angelborg and Engström, 1972; Slepecky and Chamberlain, 1987). Tightly packed MT bundles in SCs provide the architectural support required to convey mechanical signals to mechano-sensitive hair cells (Patuzzi, 1996). Although variation in MT arrangements in different cell types is believed to be essential for their morphogenesis, very little is known about MT organization during organ of Corti development.

Formation of the organ of Corti requires the assembly of complicated intercellular connections and dramatic structural changes in MTs. Although the protein networks in hair cells have been studied, we know little about protein expression in SCs. Besides the commonly observed centrosomal-based MT organization centers (MTOC, Fig. 1B, circles), each SC contains two noncentrosomal, membrane-associated MTOCs (Tucker et al., 1998; Mogensen et al., 2002; Moss et al., 2007): basal noncentrosomal MTOCs are shown by triangles while more apical noncentrosomal MTOCs are indicated by rectangles in Fig. 1B. Both noncentrosomal MTOCs display a meshwork of dense fibrous material located between adhesive junctions and MT bundles. Little is known about the protein components present in the ‘meshwork’ of these non-centrosomal MTOCs (Slepecky and Savage, 1994). It is believed, however, that the three MTOCs inside each SC (two noncentrosomal and one centrosomal) collaborate to construct/organize MT bundles and coordinate the connection of these MT bundles via adhesive junctions to neighboring cells and/or to the basilar membrane (Tucker et al., 1992; Henderson et al., 1994). Unfortunately, the details of the underlying mechanisms remain ill defined.

We previously discovered a *de novo* gene similar to Kiaa1543 (Nagase et al., 2000) from a mouse cochlear library (Zheng et al., 2009). Because mouse Kiaa1543, RIKEN 2310057J16 (NM_027171), is capable of dramatically organizing or marshaling certain proteins into distinct arrangements, we named this protein marshalin (Zheng et al., 2008; an abstract of ASCB). Marshalin was independently discovered and named by other groups as nezha (Meng et al., 2008), CAMSAP3 (Baines et al., 2009), and patronin (Goodwin and Vale, 2010). Marshalin exhibits multiple protein interacting domains including a CKK domain (DUF1781) at the C-terminus, a calponin homology (CH) domain at the N-terminus, and three coiled-coil (CC) and two proline-rich (PR) domains in the middle (Fig. 1C). It is known that CAMSAP3 binds to the minus-end of MTs through its CKK domain (Baines et al., 2009), and that marshalin's *Drosophila* homolog (patronin) regulates MTs by protecting MT-minus-ends (Goodwin and Vale, 2010). Marshalin also plays an important role at intercellular junctions as demonstrated in the epithelial cell line Caco2 (Meng et al., 2008). As a rare MT minus-end binding protein carrying several protein-protein interacting domains, marshalin may have a significant biological impact on the regulation of the cytoskeleton and on cell-to-cell communication in many different tissues. However, research regarding mammalian marshalin has been limited to cultured cells (nezha; Meng et al., 2008) and *in vitro* systems (CAMSAPs; Baines et al., 2009). In order to understand marshalin's role *in vivo* where the correct three-dimensional organization of epithelial tissues is maintained, we selected the mammalian cochlea for investigation because there are dramatically different MT patterns in HCs and SCs and the organ of Corti exhibits sophisticated cell junctions where marshalin may play important roles.

## Results

### Marshalin protein expression during cochlear development

In order to investigate marshalin protein expression in cochleae, we raised antiserum against the C-terminus of mouse marshalin. The specificity of the antibody was tested by several methods including Western blot, ELISA, and immunofluorescence (IF) as described before (Zheng et al., 2011). The coding region of marshalin (Fig. 1C) was inserted into a pcDNA6/V5HisB vector to attach a V5-His tag at the C-terminus of marshalin. Specificity of anti-marshalin was tested in transiently transfected opossum kidney (OK) cells with a plasmid encoding marshalin-V5-His cDNA. As shown by the immunofluorescent images in supplementary material Fig. S1A, similar staining patterns were observed for both anti-V5 and anti-marshalin. In addition, both anti-V5 and anti-marshalin recognized the same protein bands in Western blots of marshalin-expressing OK cells (supplementary material Fig. S1B). Furthermore, the protein bands recognized by anti-marshalin disappeared when marshalin-expressing OK cells were co-transfected with siRNA specifically targeting marshalin mRNA (supplementary material Fig. S1C). These data suggest that anti-marshalin specifically recognizes marshalin protein.

**Fig. 2.**
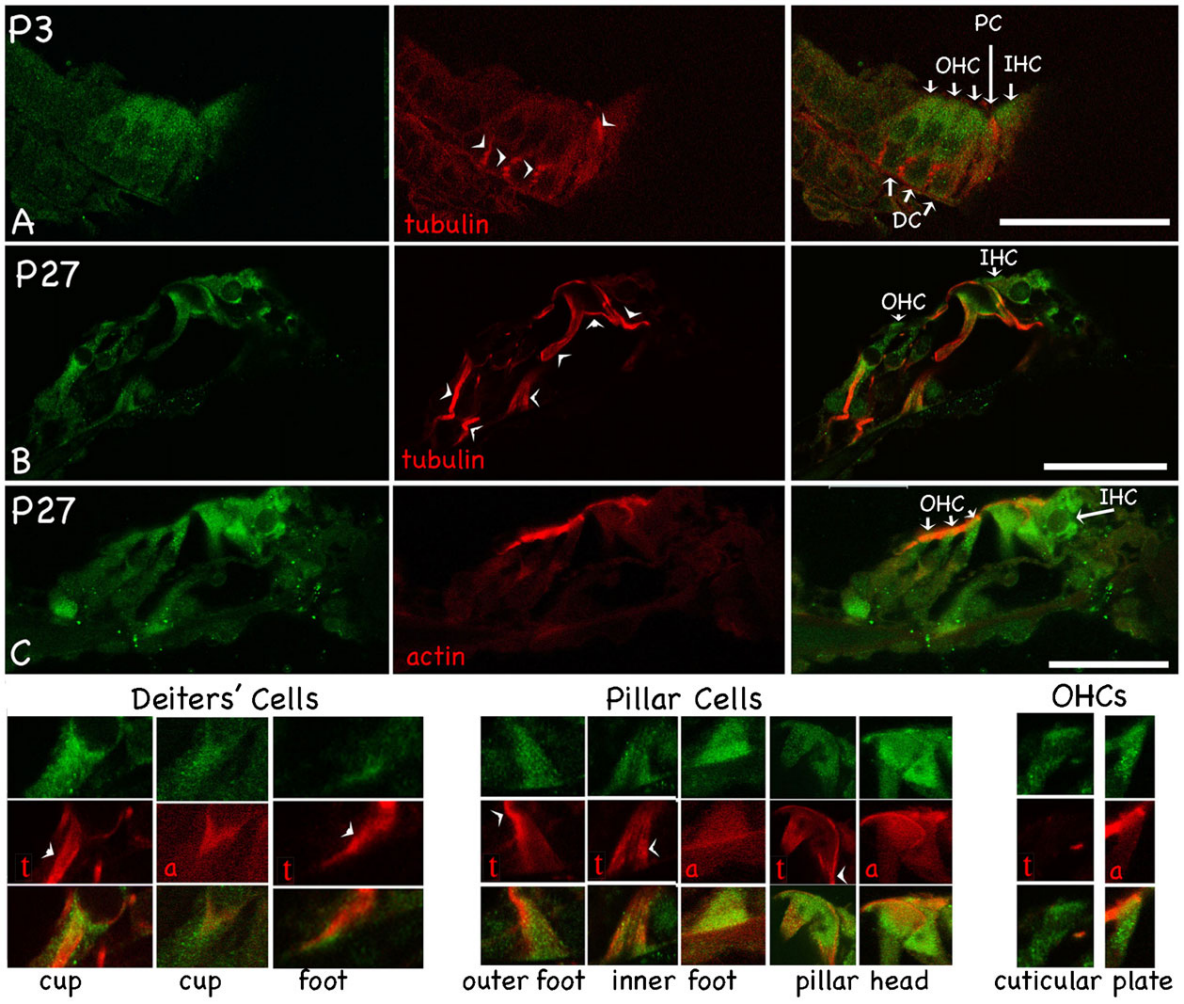
Immunofluorescent images show protein distribution for marshalin (anti-marshalin, green), MΤ (indicated by red ‘t’ or ‘tubulin’), and actin-filaments (indicated by red ‘a’ or ‘actin’). (A–B) Organ of Corti derived at P3 (A) and P27 (B) with marshalin/tubulin staining, and a merged image showing both. (C) Organ of Corti derived at P27 with marshalin, actin, and a merged image showing marshalin and actin staining. Enlarged images show the co-localization of marshalin with tubulin or actin at the cup and foot of a Deiters' cell, the feet and heads of pillar cells, and the cuticular plate of an OHC. Scale bar: 24 µm.

Detection of immunofluorescence was observed in mouse cochlea cross sections collected at different developmental stages. The results show that detectable marshalin protein is restricted to the organ of Corti and increases during development (supplementary material Fig. S2). At P0–P3, low intensity marshalin staining was observed, consistent with the fact that no or very few MT bundles are formed at this time. Nevertheless, some weak marshalin staining is found around the organ of Corti region as demonstrated in supplementary material Fig. S2 by white bars. When compared to other cells in the inner ear, cells in the organ of Corti have stronger marshalin staining from P0 to adult. As more MT bundles are formed, stronger marshalin staining is observed (supplementary material Fig. S2; Fig. 6E). At higher magnification, as shown in panel A of Fig. 2 at P3, marshalin staining was stronger in HCs than in SCs. Later in development at P27, hair cells exhibit strong marshalin staining in the cuticular plate (CP) and the cytoplasm, where it is widespread (Fig. 2B,C, OHCs). IHCs show stronger marshalin labeling than OHCs (Fig. 2B,C), reminiscent of our electron microscopy (EM) data (Furness et al., 1990) where MTs are more prevalent in IHCs. The strong marshalin staining observed in the CPs of both IHCs and OHCs appears to overlap with that for actin filaments, as judged by merged images (Fig. 2B,C, OHCs).

In SCs, MT-bundles are shown by anti-α-tubulin red staining (labeled with ‘t’ or ‘tubulin’ in Fig. 2, and in 3-D supplementary material Movie 1) in order to compare their distribution with that for marshalin. The latter's green staining is predominantly detected in the heads/feet of pillar cells, and in the cup and the foot of the Deiters' cells (Fig. 2, Deiters' cells, pillar cells). In other words, marshalin is enriched in all noncentrosomal MTOCs including basal and higher-level MTOCs (Fig. 1B). Data in Fig. 2 also demonstrate that actin microfilaments (MFs) are abundantly expressed in noncentrosomal MTOCs in SCs. Red actin filaments (labeled with ‘a’ or ‘actin’ in Fig. 2) co-localize with marshalin in many places including pillar heads and feet, Deiters' cup and foot (Fig. 2, Deiters' cells, pillar cells). In addition, marshalin protein appears to be close to the plasma membrane in the Deiters' cup and foot, and pillar cell feet and heads (Fig. 2). We also noticed that green marshalin staining spreads beyond that for red MT bundles (arrowheads) in SCs (Fig. 2, Deiters' cells, pillar cells).

**Fig. 3.**
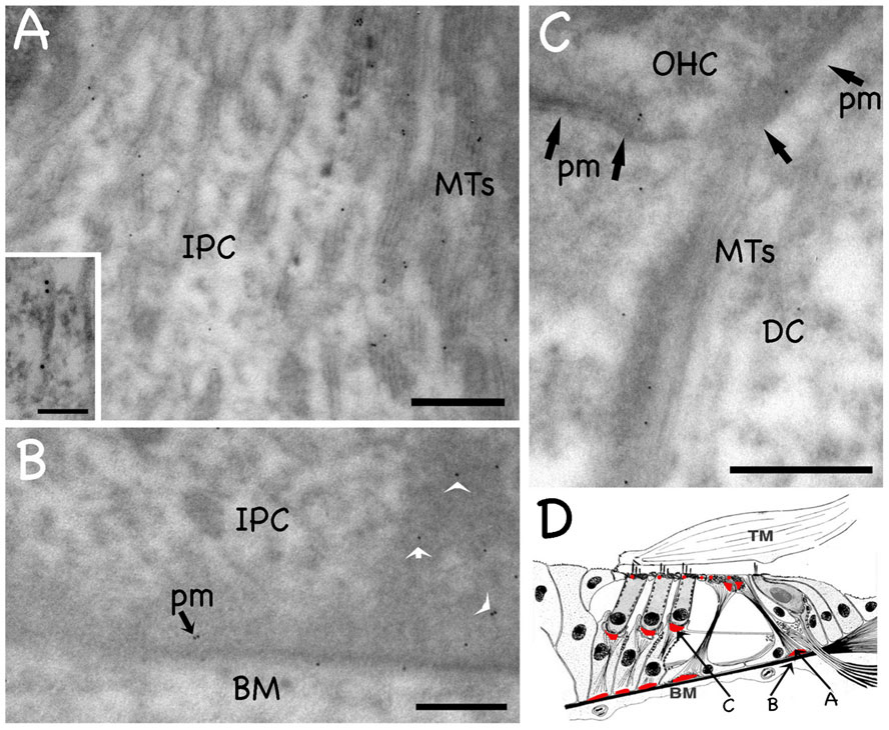
Transmission electron microscopy (EM) of post-embedding immunogold labeling for marshalin. (A) A region of the inner pillar cell (IPC) foot showing the end of the main MT bundle. MTs tend to splay out into small groups in this region, and the gold label is associated with these groups. Inset: Gold labeling associated with individual MTs. (B) In an image taken below the in panel A, gold labeling (black arrow) is shown along the plasma membrane (pm) of the inner pillar cell (IPC), which rests on the basilar membrane (BM). White arrows indicate marshalin-associated gold particles in noncentrosomal MTOCs. (C) Deiters' cell cup at the base of an OHC showing a MT bundle in the center of the image. Gold particles are associated with the MT and in the region where the base of the OHC joins the underlying Deiters' cup. The adjacent membranes are indicated by black arrows. (D) Schematic showing the approximate location of each image. Red circles indicate the locations of centrosomal-based MTOCs, red ovals/triangles show noncentrosomal MTOCs. Scale bars (A,B,C) = 500 nm; inset = 100 nm. The image shown in panel B was subjected to unsharp mask filtering (radius 2.2 pixels, amount 50%) using Adobe Photoshop CS software. As a negative control for false positive labelling, we also used anti-CHaT antibody along with the same secondary tagged with gold articles. Anti-CHaT recognizes choline acetyl transferase but does not label MTs (not shown here).

MT bundles, composed of thousands of individual MTs, are shown in SCs in Fig. 2. In order to avoid saturation, we must decrease laser power to obtain these images. It is likely that marshalin is associated with individual MTs whose fluorescent intensities are too weak to be seen by light microscopy due to the fact that the laser power was decreased to avoid saturation. As an alternative, we performed post-embedding immunogold EM. The location of images in panels A, B, and C of Fig. 3 is provided in the schematic shown in part D. Note that small groups of MTs splay out from the main MT bundle at the foot of the IPC (panel A). Although marshalin-associated gold particles are positioned around the MT bundle, as well as the individual MTs that spread out and away from it (Fig. 3A, inset), they also localize to the meshwork of dense fibrous material between the PM and MT bundles (white arrows, Fig. 3B). Marshalin-associated gold particles are also observed at the plasma membrane (indicated by black arrows in Fig. 3B,C), suggesting that marshalin is involved in building and maintaining adhesive junctions between SCs and OHCs, as well as between SCs and the basilar membrane. As a negative control, we used anti-CHaT as a mock primary antibody, which recognizes protein choline acetyl transferase. Anti-CHaT does not label MTs even though the same secondary antibody (tagged with gold particles) was used (data not shown). We further analyzed gold particle distribution in two sections of a pillar cell foot and divided this area into three regions: an MT-rich area (61% of the total measured area), the cytoplasm (22% of the total measured area), and the dense region (17% of the total measured area) that surrounds the non-centrosomal MTOC. The normalized relative densities of the gold particles found in the three distinct regions are 0.46, 0.18, and 0.36 for the MT-rich area, the cytoplasmic area, and the dense region, respectively. These data suggest that marshalin is associated with individual MTs and may also be a component of the meshwork found in connection with noncentrosomal, membrane-based MTOCs in the supporting cells. This latter possibility is consistent with the greater immunofluorescence (Fig. 2) of MTOCs, compared with that along the MT bundles.

### Identifying marshalin isoform expression in the cochlea

**Fig. 4.**
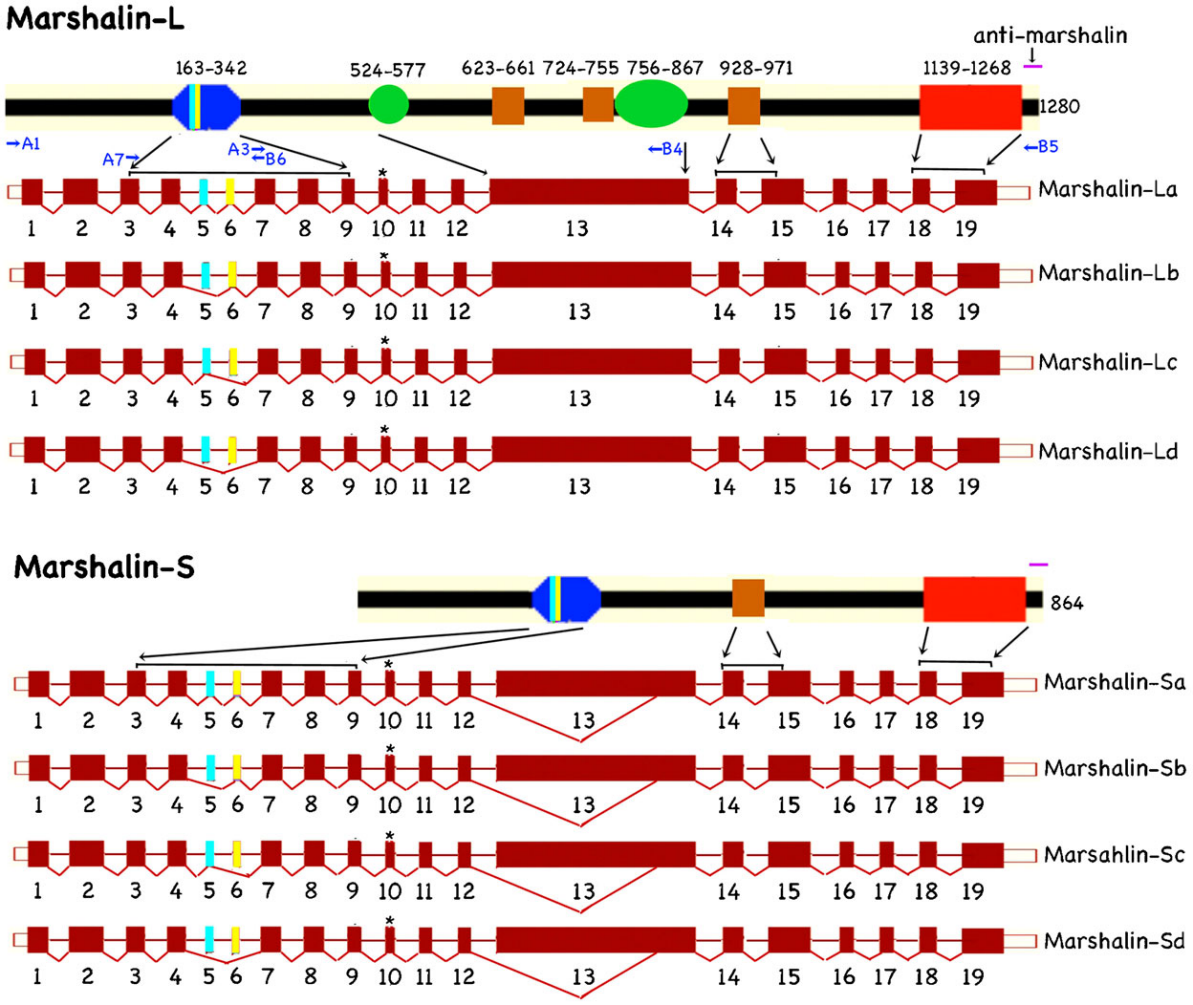
Cartoon representing mouse marshalin isoforms, alternative splice variants, and their corresponding protein domain organization. The long (L) and short (S) forms are defined by the alternative 3′ splice site in exon 13, which codes two CC domains (brown) and two PR regions (green). Marshalin-S is missing 1248 bp in exon 13. The longest marshalin-L, marshalin-La, has 1280 aa, while the longest marshalin-S, marshalin-Sa, has 864 aa. The four sub-isoform classes are designated with a letter suffix and defined by the presence or absence of two newly discovered exons: exon 5 and exon 6. Their amino acids are labeled light blue and yellow. Sub-isoform ‘a’ carries both exon 5 and exon 6 for a total of 19 exons, ‘b’ carries only exon 5, ‘c’ carries only exon 6, and ‘d’ carries neither. Additional alternative splicing at exon 10 creates variants v1 and v2 (labeled with *), with v1 being one aa longer than v2. CKK (red) is coded by exons 18 and 19. The CH (blue) is coded by exons 3 to 9. The purple line indicates the epitope location for anti-marshalin used in this study. Blue arrows indicate the positions of primers pairs: A1/B5, A7/B6, and A3/B4.

The organ of Corti is a unique system where MTs are present in loose dynamic networks in HCs and tightly packed stable bundles in SCs. Because of these structural differences, we suspected that different marshalin isoforms could be involved. Therefore, mRNA was isolated from mouse cochleae to investigate whether different marshalin isoforms are expressed. We identified 8 different *marshalin* cDNA isoforms, with each isoform falling into one of two variant categories, v1 or v2, based on alternative splicing at exon 10. Compared with v2, v1 has one additional amino acid (aa). The accessory numbers for each isoform and their variants are listed in supplementary material Table S1. Based on length, we divided *marshalin* isoforms into long (L) and short (S) forms. Marshalin-L and marshalin-S have alternative 3′ splice sites resulting in a 1248 bp deletion at the N-terminus of the largest exon, exon 13. This results in a shorter variant of the protein, marshalin-S, which is 416 aa shorter than the long variant, marshalin-L. Although the N-terminal side of *marshalin-S*'s exon 13 is not flanked by an intronic sequence, the splice junction sites of the deleted fragment are flanked by sequences that fit the GU-AG rule, thereby ensuring the formation of a mature mRNA. As shown in Fig. 4, the longest marshalin, marshalin-La, has 1280 aa encoded by 19 exons instead of 17 as previously reported (Meng et al., 2008). The newly discovered exons 5 and 6 are 33 bp and 48 bp, respectively. Marshalin isoforms containing both exons 5 and 6 are named sub-isoform ‘a’, while isoforms with only exon 5 are named ‘b’; those with only exon 6 are ‘c’, and those lacking both exons 5 and 6 are ‘d’. *Marshalin-Ld* has the same DNA sequence as nezha (NM_027171), which also lacks exons 5 and 6. *Marshalin-Lb* and *marshalin-Lc* carry either exon 5 or exon 6, but not both. These two isoforms have 18 exons with 1269 and 1264 aa, respectively. Similar to *marshalin-L*, *marshalin-S* also has 4 different sub-isoforms: *marshalin-Sa*, *-Sb*, *-Sc*, and *-Sd*. Since these isoforms have never been reported, the sequences have been deposited in Genbank with the assigned numbers listed in supplementary material Table S1. All nuclear splice junctions follow the GU-AG rule.

Marshalin has several protein-protein interacting domains according to bioinformatic analysis using programs including SMART, Prosite, ProtParam and UniProt. As shown in Fig. 4, all isoforms contain a tubulin-binding domain, CKK (red), coded by exons 18–19 at the C-terminus, and a potential actin-binding CH domain (blue) coded by exons 3 through 9 at the N-terminus. The difference among the four sub-isoforms *a*, *b*, *c*, and *d*, is the amino acid sequence in the CH domain (blue). The ‘*a*’ sub-isoform has the longest CH domain because it contains exons 5 and 6, while ‘*d*’ has the shortest because these exons are not present. Notice that marshalin-L and marshalin-S have different protein-protein interacting domains in the middle region. Marshalin-L has three CC domains and two PR regions, while marshalin-S has only the one CC domain due to the loss of 416 aa in exon 13. The latter may result in different protein/protein interactions. Anti-marshalin (purple line in Fig. 4) used in this report recognizes all isoforms.

### Different mRNA isoform expression during organ of Corti development

**Fig. 5.**
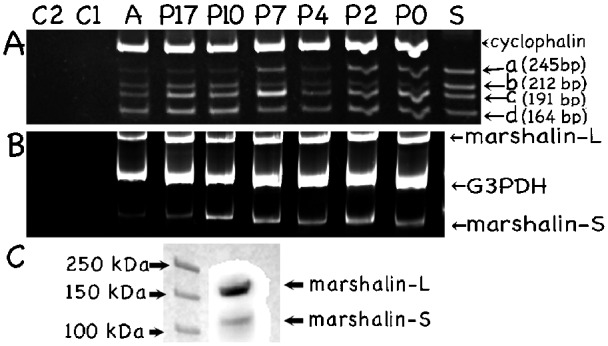
Expression of *marshalin* sub-isoforms during organ of Corti development. RNA was isolated from single litters and collected at P0, P2, P4, P7, P10, P17, and adult (A). (A) RT-PCR using marshalin A7/B6 primers with expected bands: a. 245 bp; b. 212 bp; c. 191 bp; and d. 164 bp. The internal control gene *cyclophilin* has a band at 301 bp. Lane C1: no reverse transcriptase negative control. Lane C2: no single stranded cDNA template negative control. Lane S: positive controls using DNA plasmids encoding *marshalin-Sa, marshalin-Sb, marshalin-Sc,* and *marshalin-Sd*. (B) RT-PCR using marshalin A3/B4 with expected bands: *marshalin-L* (∼1800 bp) and *marshalin-S* (∼600 bp). The internal control gene *G3PDH* (glyceraldehyde 3-phosphate dehydrogenase) has a band around 900 bp. (C) Expression of marshalin proteins was analyzed by SDS-PAGE/Western blot. Marshalin-L and marshalin-S bands were identified in a P21 cochlea using anti-marshalin.

It is common for different mRNA isoforms to appear at different times in cochlear development (Lagziel et al., 2005; Ahmed et al., 2006). To investigate marshalin's role during organ of Corti development, RNA samples were collected from mice at E17, P0, P2, P4, P7, P10, P17, and adult. *Marshalin* mRNA has a high GC content with abundant short direct repeat sequences, which can result in false alternative transcripts during cDNA synthesis through reverse transcriptase (Cocquet et al., 2006). Therefore, we used a thermostable reverse transcriptase to minimize this complication. RT reactions were also performed at 55°C instead of the standard 42°C. As shown in Fig. 5A, sub-isoforms, *a*, *b*, *c* and *d*, were detected using marshalin A7/B6 primers (blue arrows in Fig. 4) in all cochlear samples collected from one litter at different developmental stages including P0 and adult. RNA corresponding to *marshalin-L* and *marshalin-S* was also detected in all samples tested by RT-PCR using marshalin A3/B4 primers (blue arrows in Fig. 4), even though the marshalin-S band is less bright in adult samples than in immature cochleae (Fig. 5B). Intensities of the marshalin-L bands are stronger than the marshalin-S bands for all samples, suggesting that more *marshalin-L* mRNA is expressed. Bands were not detected in the control conditions where reverse transcriptase (C1) or single stranded cDNA templates (C2) were omitted.

Expression of marshalin proteins was also analyzed by SDS-PAGE/Western blot. We observed two protein bands recognized by anti-marshalin in P21 cochlear samples (Fig. 5C). These bands are near 150 kDa and 100 kDa respectively, similar to the band sizes found by Western blot for marshalin-Ld-V5-His and marshalin-Sd-V5-His proteins synthesized in OK cells through transient transfection. As for the mRNA data shown in Fig. 5B, the intensity of marshalin-L is stronger than marshalin-S, suggesting that more *marshalin-L* mRNA and protein is expressed in adult cochleae.

### Marshalin induces MT-based bundle formation

**Fig. 6.**
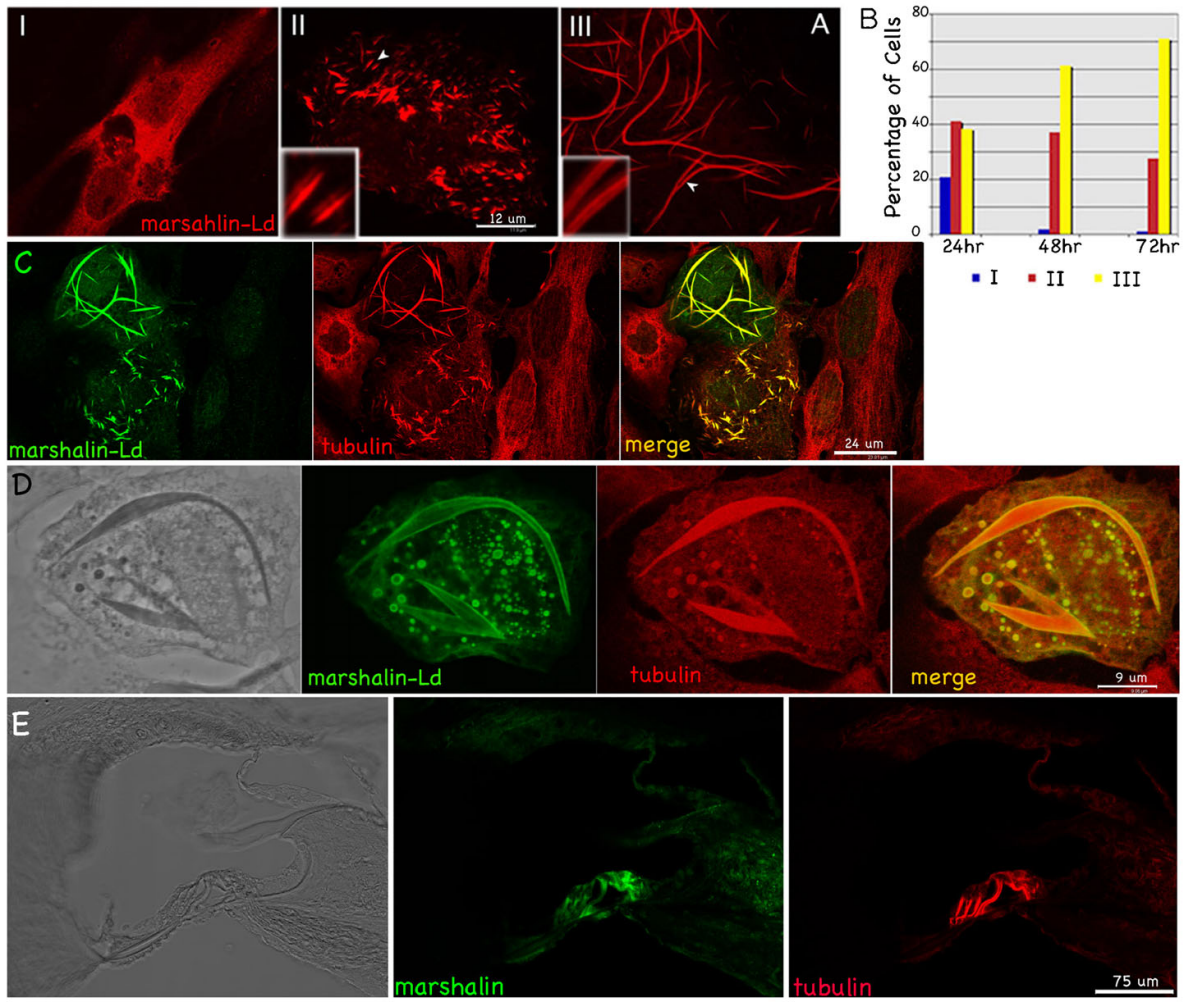
Marshalin-Ld re-organizes MT networks. (A) Three different marshalin protein expression patterns in OK cells. Marshalin was detected with anti-V5 (red). For better visualization, portions of ‘stick’ and ‘string’ formations (indicated by arrowheads) are shown in the left corners at higher magnification. (B) Three types of expression patterns at 24 hrs, 48 hrs and 72 hrs post transfection with plasmids encoding *marshalin-Ld-V5-His*. (C) Co-localization of marshalin-Ld and MTs in OK cells transfected with a plasmid encoding marshalin-Ld-V5-His. (D) MTs form the “core” structure of stick/string formations while marshalin staining appears along the outer boundaries. The image on the far left is the phase-contrast image. (C–D) Marshalin detected with anti-V5 (green); MTs with anti-α-tubulin (red) 48 hr post transfection. (E) Marshalin protein expression in the young adult inner ear. Anti-marshalin immunofluorescent image (green) was taken at low magnification to emphasize that expression dominates in the organ of Corti, where strong MT staining (anti-α-tubulin, red) was also observed. The corresponding bright-field image of the inner ear is shown on the left. Scale bars: 12 µm (A), 24 µm (C), 9 µm (D), 75 µm (E).

To study the function of various marshalin isoforms, marshalin-Ld and marshalin-Sd were selected for further investigation. As shown in Fig. 6E, strong marshalin signals (green) were only visible in the organ of Corti area where strong MT bundles (red) were also found. Most cells located outside the organ of Corti have little marshalin signal. In fact, mean fluorescent intensities of marshalin staining in the cuticular plate of hair cells and the noncentrosomal MTOC areas in supporting cells are more than 10 times higher than those in cells outside the organ of Corti. Based on this observation, we speculate that increasing marshalin expression in the organ of Corti during development is directly related to the gradual formation of the highly bundled MT structures that characteristic of the pillar cells and Deiters' cells of the organ of Corti in the inner ear.

Marshalin-Ld and marshalin-Sd were selected for further investigation in a heterologous expression system in order to study isoform-specific effects of marshalin on MT bundle formation. When OK (opossum kidney) cells were transiently transfected with plasmids encoding *marshalin-Ld-V5-His*, three different expression patterns were observed. As in Fig. 6A, Type I patterns (upper left) show a typical protein expression arrangement with marshalin staining throughout the cytoplasm. Type II patterns are made up of short ‘sticks’, while those for type III appear as long ‘strings,’ some of which are over 50 µm in length. Both ‘stick’ and ‘strings’ have two layers of marshalin protein staining as shown at high magnification. In OK cells, the distribution of these three patterns of protein expression appears to depend on marshalin concentration. As shown by the bar graph in Fig. 6B, 24 hrs after *marshalin-Ld* transfection, 22% of marshalin-expressing cells were type I, 42% were type II, and 38% were type III. However, 71% of cells were type III after 72 hrs while only 1% were type I, implying that as more marshalin protein is synthesized, a higher percentage of type III patterns is observed. In other words, the degree of MT bundle formation seems to correlate with the degree of marshalin expression.

We also examined cells transfected by different marshalin-Ld constructs: *marshalin-Ld*, V5-His-tagged *marshalin-Ld*, and GFP-tagged *marshalin-Ld*. Expression of these versions of marshalin created similar intracellular structures (type II and III) (supplementary material Fig. S3). In other words, the addition of V5, His or GFP tags at the C-terminus of marshalin did not affect marshalin-induced bundle formation. As shown in Fig. 6C and supplementary material Fig. S4A, marshalin (green) staining co-localizes with tubulin (red) staining, i.e., MTs in marshalin-expressing cells were modified into extended ‘string’ or ‘stick’ shapes. In contrast, cells without marshalin show a regular MT network without sticks/strings as shown in Fig. 6C (red staining). Fixed cells with methanol or formaldehyde in either PBS or a MT stabilizing buffer (MTSB) also showed similar marshalin staining patterns, suggesting that these marshalin-induced bundles are not artifact. Some ‘strings’ in marshalin-expressing OK cells expand to form spindle-shaped tubes, showing a dense outline with phase contrast (Fig. 6D, far left). Tubulin appears in the lumen, while marshalin forms the outer layers. Taken together, these data indicate that marshalin-induced bundles are MT-associated.

We also treated marshalin-expressing cells with colchicine and paclitaxel, which stimulate MT disassembly and assembly, respectively. Paclitaxel does not change MT bundle formation significantly (supplementary material Fig. S4C). However, colchicine-treated cells show type I distribution with diffused marshalin and tubulin staining throughout the cytoplasm (supplementary material Fig. S4B). In other words, all type II (stick) and type III (string) cells disappeared when treated with colchicine (1 µg/µl for 3 hrs or 250 ng/µl 24 hrs). In contrast, marshalin-Ld-induced bundles still emerged in the presence of chemicals that modify actin filaments such as jasplakinolide (10 nM for 24 hrs, supplementary material Fig. S4D), cytochalasin D (0.5 µM for 24 hrs, supplementary material Fig. S4E), and latruculin A (0.25 µM for 24 hrs, supplementary material Fig. S4F). Under these conditions, most actin filaments were disassembled after treatment (supplementary material Fig. S4G). These data suggest that tubulin assembly is essential for the formation of marshalin-associated bundles.

**Fig. 7.**
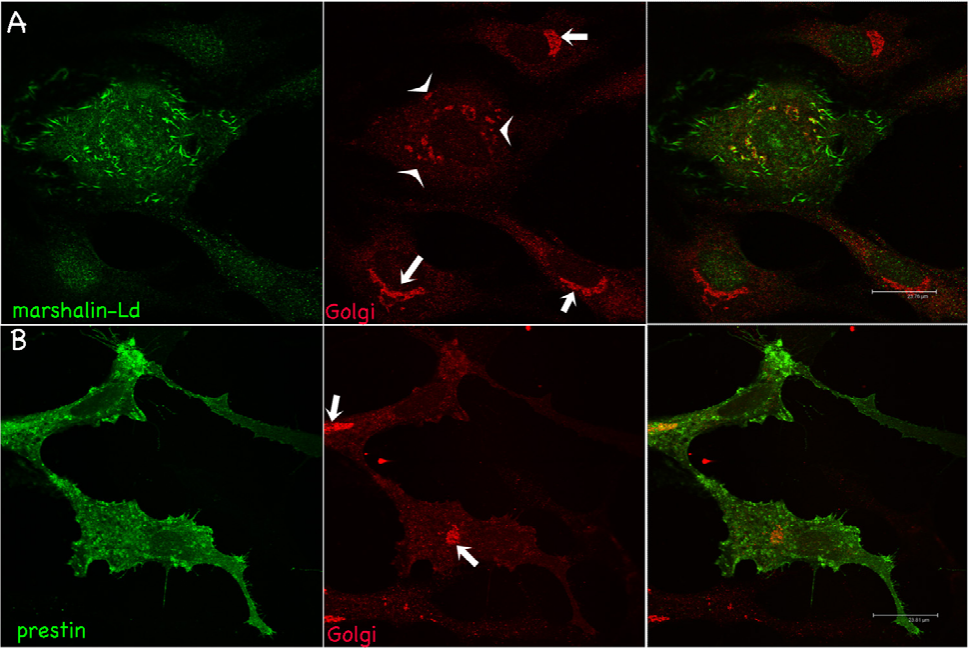
Marshalin's relationship with the Golgi apparatus. OK cells were transfected with plasmids encoding *marshalin-Ld-V5* (A) and *prestin-GFP* (B), respectively. Marshalin (prestin) was stained with anti-marshalin (anti-prestin) (green); Golgi with anti-Golgin 97 (red). Arrows indicate normal Golgi structures near nuclei. Arrowheads show Golgi fragments distributed in cytoplasm. Scale bars: 24 µm.

We then examined other membranous organelles in marshalin-expressing cells. The Golgi network, normally located near the nucleus, is disrupted and separates into fragments in cells expressing marshalin-Ld (Fig. 7A, arrowheads). These Golgi-membrane fragments co-localize with marshalin protein (green). Such fragmentation was not seen by overexpression of other proteins such as GFP and prestin (Fig. 7B, arrows), the OHC-specific motor protein (Zheng et al., 2000). Because the location and distribution of the Golgi network is closely connected with the cytoskeleton (Sandoval et al., 1984), Golgi disruption may be due to dramatic changes in MTs caused by marshalin-Ld expression.

### Marshalin-L and marshalin-S isoforms induce different MT-bundle structures

**Fig. 8.**
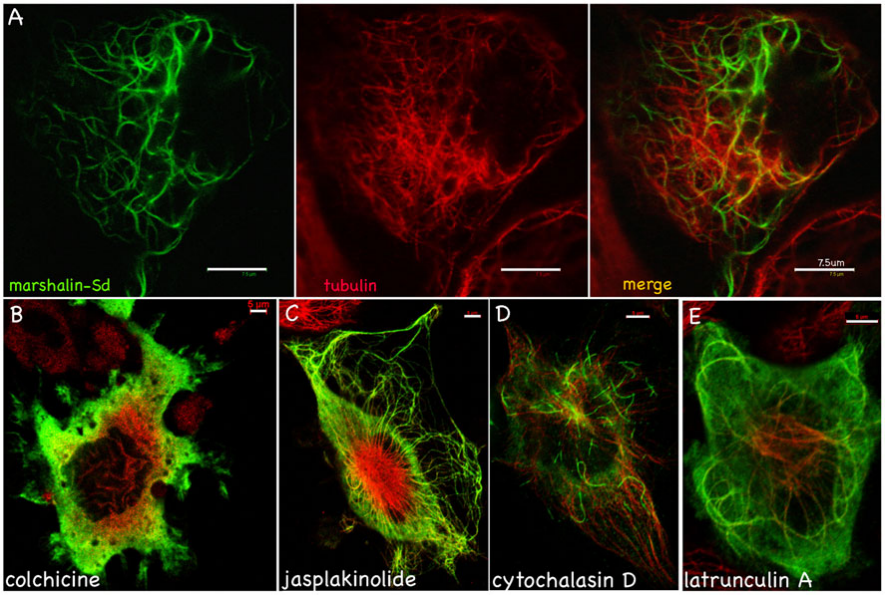
MT bundles induced by *marshalin-Sd* cDNA. Marshalin-Sd-V5-His-expressing OK cells stained with anti-V5 for detecting marshalin (green) and anti-α-tubulin for detecting MTs (red) and merged images showing both 48 hrs post transfection. (A) Cells without drug treatment. Scale bar: 7.5 µm. (B) Cells treated with 250 ng/µl colchicine, a microtubule polymerization inhibitor, for 24 hrs. (C) Cells treated with 10 nM jasplakinolide, which increases actin filament assembly, for 24 hrs. (D) Cells treated with 0.5 µM cytochalasin D, which inhibits actin polymerization, for 24 hrs. (E) Cells treated with 0.25 µM latruculin A for 24 hrs, which also inhibits actin polymerization. Panels B–E are merged images showing both MT and marshalin-Sd staining. Scale bars: 5 µm (B–E).

Marshalin-L and marshalin-S differ by 416 aa, which include protein-protein interaction domains CC and PR. When *marshalin-Sd* cDNA was expressed in OK cells, MT bundles were also induced. However, the marshalin-Sd associated MT bundles were different from those induced by marshalin-Ld. As shown in Fig. 6A, marshalin-Ld induced thick bundles with double marshalin-staining borders, lying side by side as indicated by arrows. In contrast, MT bundles induced by marshalin-Sd lack double layers (Fig. 8A). Marshalin-Sd-associated MTs bundles are more slender and curled than those induced by marshalin-Ld. Marshalin-Sd associated MTs appear integrated into MT woven networks. In addition, the spindle-shaped tubes (Fig. 6D), which are often observed in marshalin-Ld-expressing cells, are not found in marshalin-Sd-expressing cells. These data suggest that bundles induced by marshalin-L and marshalin-S have different structures and/or compositions.

We also treated marshalin-Sd-expressing cells with chemicals that modify MTs and actin filaments. As shown in Fig. 8B, colchicine-treated cells show no marshalin-Sd-associated bundles, i.e., marshalin and tubulin staining appear throughout the cytoplasm. These data suggest that tubulin assembly is also essential for the formation of marshalin-Sd associated bundles. Similar to marshalin-Ld-expressing cells, marshalin-Sd induced bundles are present in all cells treated with chemicals that modify actin filaments including jasplakinolide (Fig. 8C), cytochalasin D (Fig. 8D), and latruculin A (Fig. 8E). For example, latrunculin A sequesters G-actin, thereby inhibiting actin polymerization. As shown in Fig. 8E, marshalin-Sd-induced bundles are still observed in marshalin-Sd-expressing OK cells after incubation with 500 nM latrunculin A for 3 hrs or 250 nM for 24 hrs. This evidence indicates that marshalin-Sd-induced bundle formation does not require actin assembly.

## Discussion

Formation of the organ of Corti requires dramatic structural changes in the cytoskeleton and assembly of complicated intercellular connections. By examining marshalin expression patterns at different developmental stages, we show that this protein is highly expressed in those cells that eventually become hair cells, Deiters' cells and pillar cells. The latter supporting cells develop dense MT bundle structures that are not seen in other mammalian cell types. Based on this observation, we speculate that increasing marshalin expression in the organ of Corti during development in these cells is directly related to the formation of unique bundled MT structures. In fact, MT bundle formation was induced in heterologous expression systems upon forced expression of marshalin to mimic the high expression levels of marshalin seen in the organ of Corti. Since distribution of other cellular components, which also affect cytoskeletal structures, varies among different cell types, it is hard to deduce the physiological role of marshalin solely from our observations in heterologous expression systems. It should be emphasized, however, that MT-bundling activity was biochemically demonstrated for CAMSAP1 (Baines et al., 2009). CAMSAP1 and marshalin (or CAMSAP3) have similar protein-protein interaction domains including CH, PR, CC and CKK, implying that MT bundling activity is shared among CAMSAP family members. In other words, the MT bundles induced by forced expression of marshalin in heterologous expression systems is relevant to formation of the unique dense MT bundle structures seen in Deiters' cells and pillar cells in the organ of Corti, which show remarkably high marshalin expression. Because few other cells exhibit MT bundles *in vivo*, marshalin's role in MT bundling may be unique to these cells in the cochlea.

It is also known that supporting cells have two noncentrosomal, membrane-based MTOCs that contain a ‘meshwork’ of dense fibrous material between cellular junctions and MT bundles. Abundant marshalin expression was documented around noncentrosomal MTOCs, i.e., at the heads of pillar cells, cups and feet of DCs, IPs, and OPs. This observation suggests that marshalin is likely a component of the meshwork associated with noncentrosomal, membrane-based MTOCs in these cells. As an MT-minus-end binding protein carrying several protein-protein interacting domains, marshalin is likely involved in the establishment and maintenance of noncentrosomal MTOCs in SCs. Unfortunately, lack of biological markers for non-centrosomal MTOCs prevents us from verifying whether abundant marshalin indeed increases non-centrosomal MTOCs.

Since dynamic cytoskeletal structural changes and construction of intercellular connections take place during organ of Corti development in both hair cells and supporting cells, it is not surprising that marshalin is abundantly expressed in these areas. However, it is unclear why so many different marshalin isoforms, eight in total, are simultaneously expressed in the organ of Corti at various times during development. These isoforms carry different protein-protein interaction domains that play key roles in building protein networks. For example, the CC domain is one of the principal oligomerization motifs found in many proteins (for a review, see Burkhard et al., 2001), and PR is often recognized by SH3, a common protein-interaction module (for reviews, see Mayer, 2001; Kaneko et al., 2008). These observations are consistent with the knowledge that MT networks can be regulated by many factors, including MT binding proteins. Many MT binding proteins have multiple interacting domains such as CC and PR, allowing them to partner with different proteins in a mutually exclusive and/or competitive fashion (for reviews, see Akhmanova and Steinmetz, 2008; Akhmanova and Steinmetz, 2010). In fact, we have found that marshalin-L and marshalin-S isoforms induce different cytoskeletal structures because they carry different protein-protein interacting domains. These data suggest that marshalin isoforms are capable of modifying cytoskeletal networks through interactions with various protein partners in different cell types. In this sense, marshalin is a scaffold protein, helping to organize the internal cytoskeleton and, subsequently, the distribution of membranous organelles.

It has also been reported that the CKK domain (from *Drosophila*) binds uniformly along the MT surface, while minus-end binding requires the cooperation of multiple regions within the entire marshalin protein (Goodwin and Vale, 2010). Using immunogold EM, we observed marshalin-associated gold particles along MTs, and at the end of MT bundles. These data support the idea that marshalin is not only a MT-minus-end binding protein, utilized for stabilizing MTs, but also a scaffold protein capable of interacting with multiple proteins. SCs and HCs express different sets of potential marshalin-interacting proteins that could bind to different domains of marshalin. For example, myosin 7a and αII spectrin have SH3 domains that may potentially target PR1 or PR2. However, myosin 7a is only expressed in HCs (Hasson et al., 1995) while spectrin is found in both hair cells and supporting cells (Mahendrasingam et al., 1998). Even for the same protein, it is commonly observed that different isoforms are expressed in different cell types in the organ of Corti. For example, there are seven isotypic forms of β-tubulin but not all isotypes are synthesized in every cell type within the organ of Corti (Jensen-Smith et al., 2003). Since different marshalin isoforms (with different protein-protein interacting domains) are expressed in the organ of Corti where HCs and SCs are known to have different potential marshalin-associated proteins, it is conceivable that marshalin may be involved in establishing and maintaining cytoskeletal networks, as well as various cell-cell/cell-basilar membrane connections. Expression of the eight *de novo* isoforms identified in this report provides a molecular basis upon which the differences in MT networks/intercellular junctions found in hair cells and supporting cells can be understood. As a MT-minus-end binding protein with multiple variants that are abundantly expressed in the organ of Corti at different developmental stages, marshalin is an important scaffold protein that may have significant implications for organ of Corti formation and thus for normal hearing.

## Materials and Methods

### DNA constructs and antibodies

Animal care and use procedures were approved by Northwestern University's Institutional Review Board and the NIH. After animals were euthanized with an overdose of anesthetic (Euthasol 200 mg/kg), cochleae were dissected in RNAlater (Qiagen) using mice between E17 and adult. RNA was isolated using the Absolutely RNA®RT-PCR Miniprep Kit (Stratagene) and RNA quality was measured by a 2100 Bioanalyzer (Agilent). Reverse transcription was performed by thermostable reverse transcriptase (Roche, Indianapolis, IN) at 55°C for one hour. *Marshalin* cDNA was cloned using forward primer A1: 5′-CGGCGTCGCCATGGTGGAAGC and reverse primer B5: 5′-AGGAGTGCACTGCCCAAGGTG. Cycling conditions were as follows: 95°C for 45 sec, 60°C for 1 min, and 72°C for 3 min. The coding region of *marshalin* was inserted into pcDNA6/V5His, pEGFP-N2, and pDsRed2-N1 vectors, thus attaching a V5-His, GFP, or RFP tag, respectively, to the C-terminus of *marshalin*. Marshalin isoform expression was investigated using two sets of marshalin primers: A7/B6 (A7: 5′-GTAGACACAACTGTTCGGCG, B6: 5′-GTCACATTTGGAAAGCAGGG), and A3/B4 (A3: 5′-CCCTGCTTTCCAAATGTGAC, B4: 5′-TAGAGGGATCTCAATGAGG). Cyclophalin and G3PDHA primers were used as internal RT-PCR controls (Zheng et al., 2000). PCR products were observed on 8–10% polyacrylamide/TBE gels. A 17-aa peptide (FTIQGHLWQSKKPTTPK) was used to immunize rabbits and to generate an affinity-purified anti-marshalin antibody (Covance, Denver, PA). Anti-marshalin was used at a final concentration of 2–19 µg/ml for immunofluorescence (IF) and immunogold EM and 1 µg/ml for Western blot. Anti-V5 (Invitrogen) was used at a 1:1000 dilution for IF, and 1:5000 for Western blot. Antibody dilutions were as follows: anti-V5 polyclonal antibody for Western blots (1:2500, Sigma, Saint Louis, MO); anti-GFP for Western blots (1:2000, Clontech, Mountain View, CA); anti-α-tubulin for IF (1:800, (Zymed, San Francisco, CA)); and anti-Golgin97 for IF (1: 400, Molecular Probes, Eugene, OR). Secondary antibodies included: goat anti-mouse IgG-Alexa Fluor 546 and goat anti-rabbit IgG-Alexa Fluor 488 (Molecular Probes, Eugene, OR); goat anti-rabbit IgG-HRP and goat anti-mouse IgG-HRP (Jackson ImmunoResearch, West Grove, PA); and goat anti-mouse IgG1-HRP (Alpha Diagnostic International). Texas Red-X phalloidin (1:2000, Molecular Probes, Eugene, OR) was used to stain F-actin.

### Cell culture and immunofluorescence (IF)

Plasmids encoding *marshalin*, V5-His tagged *marshalin* or GFP-tagged *marshalin* were transiently transfected into OK as previously described (Zheng et al., 2001). In some cases, siRNA for marshalin (Ambion, Grand Island, NY) was co-transfected with plasmids encoding *marshalin* cDNA. Approximately 24–45 hours post transfection, cells were treated using the following chemicals from Calbiochem: paclitaxel at a concentration of 0.38 µM for 24 hrs or 0.5 µM for 3 hrs; colchicine at a concentration of 250 ng/ml for 24 hrs or 1 mg/ml for 3 hrs; jasplakinolide at a concentration of 10 nM for 24 hrs; latrunculin A at a concentration of 250 nM for 24 hrs or 500 nM for 3 hrs; and cytochalasin D at 500 nM for 24 hrs. Chemically treated and untreated cells were fixed with 1–2% formaldehyde in PBS for 10 minutes. Cells were incubated with monoclonal anti-V5 or rabbit polyclonal anti-marshalin for 1 hr, following by incubation with secondary antibodies, goat anti-mouse IgG-Alexa Fluor 546 or goat anti-rabbit IgG-Alexa Fluor 488. In some samples, cells were first washed with warm (32–37°C) PBS, and then incubated with warm MT stabilizing buffer (MTSB) for 5 min, before being fixed in 1–2% formaldehyde/MTSB for 10 minutes. MTSB is known to stabilize MTs and to enhance the extraction of soluble proteins. MTSB contains 100 mM Pipes, pH 6.8, 1 mM EGTA, 1% Triton X-100, and 4% polyethylene glycol.

### Immunofluorescence for cochlear samples

The procedure for cochlear IF has been previously described in detail (Homma et al., 2010). Briefly, anesthetized mice were cardiac perfused first with 37°C Cytoskeleton Buffer with sucrose (CBS) (10 mM MES, pH 6.1, 138 mM KCl, 3 mM MgCl_2_, 2 mM EGTA, 300 mOsm adjusted by sucrose), and then with 4% formaldehyde (EM grade)/CBS. Following a 2 hour post-fixation at room temperature, cochleae were placed in 10% EGTA/PBS at 4°C overnight. Decalcified samples were placed in 30% sucrose/PBS and embedded in cold OCT. Organs of Corti were cut in 10–20 micron sections, placed on glass slides, fixed in 4% formaldehyde for 10 minutes and blocked at room temperature for 30 minutes in blocking solution (0.5% BSA, 5% goat or donkey serum, 0.3% Triton X-100 in PBS). Samples were then incubated with anti-marshalin or anti-α-tubulin followed by incubation with anti-rabbit-IgG/anti-mouse IgG conjugated with AlexaFluor-488, AlexaFluor-546 or Texas Red-X phalloidin. Samples were observed using a Nikon C2 spectral laser scanning confocal and a Leica confocal system with a standard configuration DMRXE7 microscope. 3-D movie and 3-D pictures were created using ImageJ.

### Post-embedding immunogold electron microscopy

Cochleae were excised from anesthetized wild-type (C3HeB/FeJ) mice and perfused with 4% freshly dissolved paraformaldehyde in 0.1 M sodium phosphate buffer through the round and oval windows, with a drainage hole in the apex. They were immersed for 2 h in fixative, then stored in fixative diluted 1:10 prior to embedding. After dissection of the cochlear wall to expose the organ of Corti, samples were dehydrated in an ethanol series and embedded in LR White resin (Agar Scientific, Stansted, UK). The embedded cochleae were bisected along a mid-modiolar plane using an annular diamond blade on a Malvern Instruments 2A micro slicer and the half block trimmed to obtain sections of the organ of Corti. Ultrathin (100 nm) sections were cut on a Reichert Ultracut F ultramicrotome and mounted on nickel grids for labeling.

Labeling was performed in drops of solution in which the grids were immersed at room temperature, unless otherwise stated, in the following sequence: wash in 0.05 M tris-buffered saline (TBS) (5 min), block in 10% goat serum in TBS (30 min), anti-marshalin antibody diluted 1:100 in TBS containing 1% goat serum (GS-TBS) overnight at 4°C, wash in TBS (3 × 5 min), goat anti-rabbit-10 nm gold conjugate (2 hrs), wash in TBS (3 × 5 min) and finally in distilled water (2 × 5 min). After removing excess water with filter paper, grids were stained in 2% aqueous uranyl acetate for 10 min, dried and examined in a JEOL JEM100SX or JEM100CX. Images were acquired using a Megaview III camera (Olympus) or a custom digital acquisition system. For negative and false positive controls, primary marshalin antibody was either omitted from the antibody incubation medium or replaced with anti-choline acetyl transferase antibody at the same dilution.

### Western blot analysis

Cochleae and OK cell samples were harvested and lysed in cold lysis buffer (50 mM Tris-HCl, pH 7.6, 150 mM NaCl, 1% Triton X-100) supplemented with a protease inhibitor cocktail (1:100) and 100 µg/ml PMSF. Insoluble material was removed by centrifugation at 3000 ×g for 10 minutes. Proteins were resolved using 4–20% SDS-PAGE, followed by immunoblotting using anti-marshalin, anti-GFP, or anti-V5 followed by anti-rabbit IgG-HRP, anti-chicken IgG-HRP, or anti-mouse IgG-HRP. Signals were detected using an ECL chemiluminescent substrate (Pierce, Rockford, IL). A Kodak Imaging System was used to capture the images as described previously (Sengupta et al., 2009).

## Supplementary Material

Supplementary Material
