# Supplementary material for: Marshalin, a microtubule minus-end binding protein, regulates cytoskeletal structure in the organ of Corti
Source: Biol Open. 2013 Sep 17;2(11):1192–202. doi: 10.1242/bio.20135603 (PMC3828766; doi:10.1242/bio.20135603)
Supplement: Supplementary Material [file supp_2_11_1192__index.html]

Marshalin, a microtubule minus-end binding protein, regulates cytoskeletal structure in the organ of Corti — Supplementary Material 

# Marshalin, a microtubule minus-end binding protein, regulates cytoskeletal structure in the organ of Corti

## bio.20135603 Supplementary Material

**Files in this Data Supplement:**

- Supplementary Material - Jing Zheng et al. doi: 10.1242/bio.20135603
- Movie 1 - **Movie 1. 3D-movie showing the location of marshalin in the organ of Corti.** Thirty individual images were collected from a P27 mouse cochlea at intervals of 0.15 µm per section. The movie was created using ImageJ.
