# Supplementary material for: Marshalin, a microtubule minus-end binding protein, regulates cytoskeletal structure in the organ of Corti
Source: Biol Open. 2013 Sep 17;2(11):1192–202. doi: 10.1242/bio.20135603 (PMC3828766; doi:10.1242/bio.20135603)
Supplement: Supplementary Material [file supp_bio.20135603_bio.20135603-s1.pdf]

Supplementary Material

Jing Zheng et al. doi: 10.1242/bio.20135603

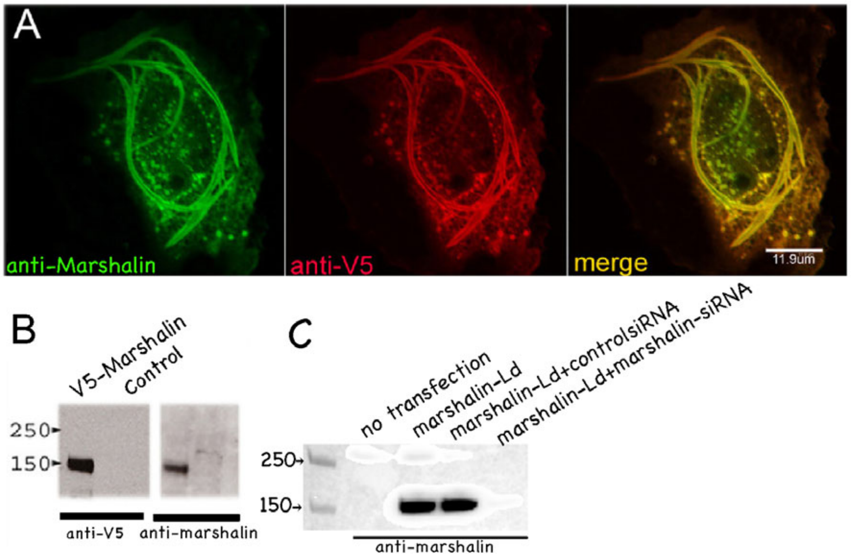

Fig. S1. Specificity of anti-marshalin. *Marshalin-Ld* (~3759 bp) cloned from the mouse cochlea was inserted into a mammalian expression vector with a V5-His epitope downstream of and in-frame with *marshalin-Ld*.
(A) Immunofluorescent images of marshalin-Ld-expressing OK cells labeled with anti-marshalin (left, green) and anti-V5 (middle, red), which recognizes the V5-tag at the C-terminus end of the marshalin protein. The right image superimposes green and red images to indicate that the staining for V5 and marshalin overlap. Scale bar: 10 µm.
(B) Western blot for marshalin-Ld-V5-His-expressing OK cells (V5-marshalin) vs untransfected cells (control). The marshalin-Ld protein band on SDS-PAGE was recognized by both anti-V5 and anti-marshalin. (C) Marshalin siRNA mediated knockdown of marshalin expression. OK cells were transfected with one of the following combinations: (1) Marshalin-Ld plasmid alone. (2) Marshalin plasmid plus negative control siRNA. (3) Marshalin-Ld plus marshalin siRNA.

Biology Open

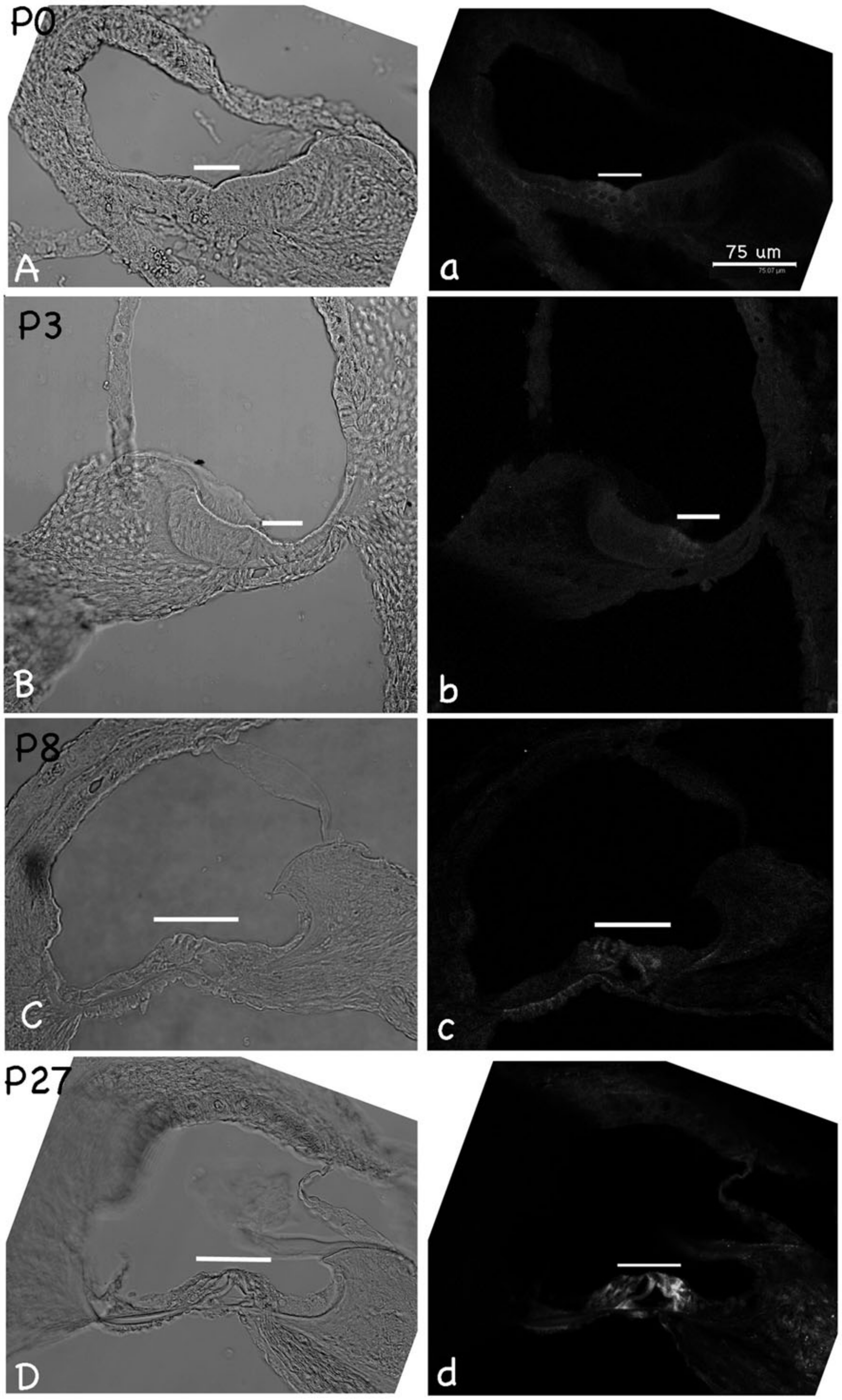

Fig. S2. Marshalin protein expression at different developmental stages. Anti-marshalin immunofluorescent images were taken at low magnification to emphasize that expression is restricted to the organ of Corti. (A) P0; (B) P3; (C) P8; (D) P27. Corresponding light microscope images appear on the left hand side. White bars overlie the OC region. Negative controls using anti-marshalin pre-incubated with antigen peptide or no anti-marshalin both lacked staining. Scale bar: 75 μm.

Biology Open

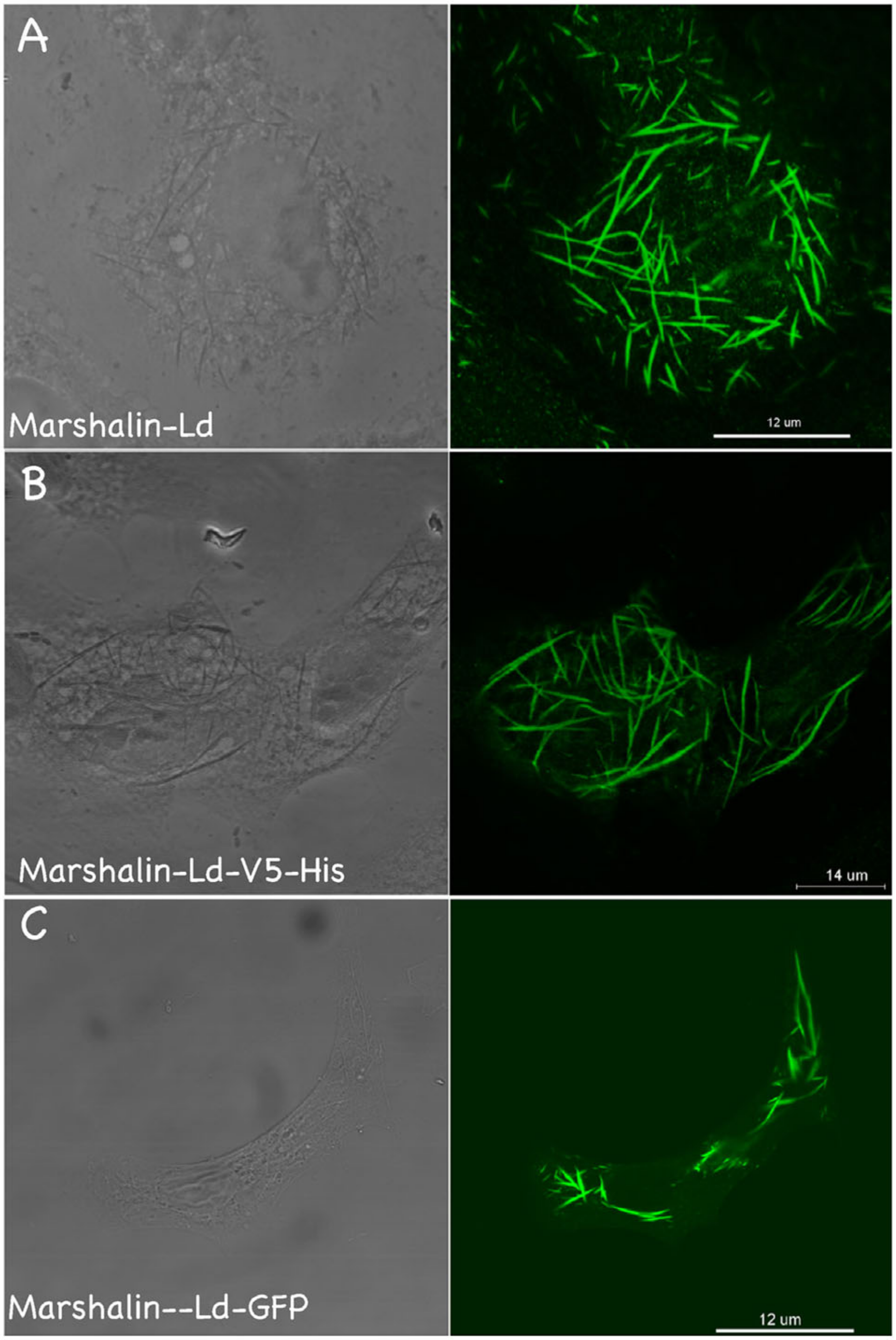

Fig. S3. V5 and GFP tags do not affect MT bundle formations. OK cells were transfected with plasmids encoding (A) *marshalin-Ld* with no tag; (B) *marshalin-Ld* with a V5-His tag; (C) *marshalin-Ld* with a GFP tag. Images were taken 48 hrs after transfection with different plasmids. Scale bars: 12 µm (A,C), 14 µm (B).

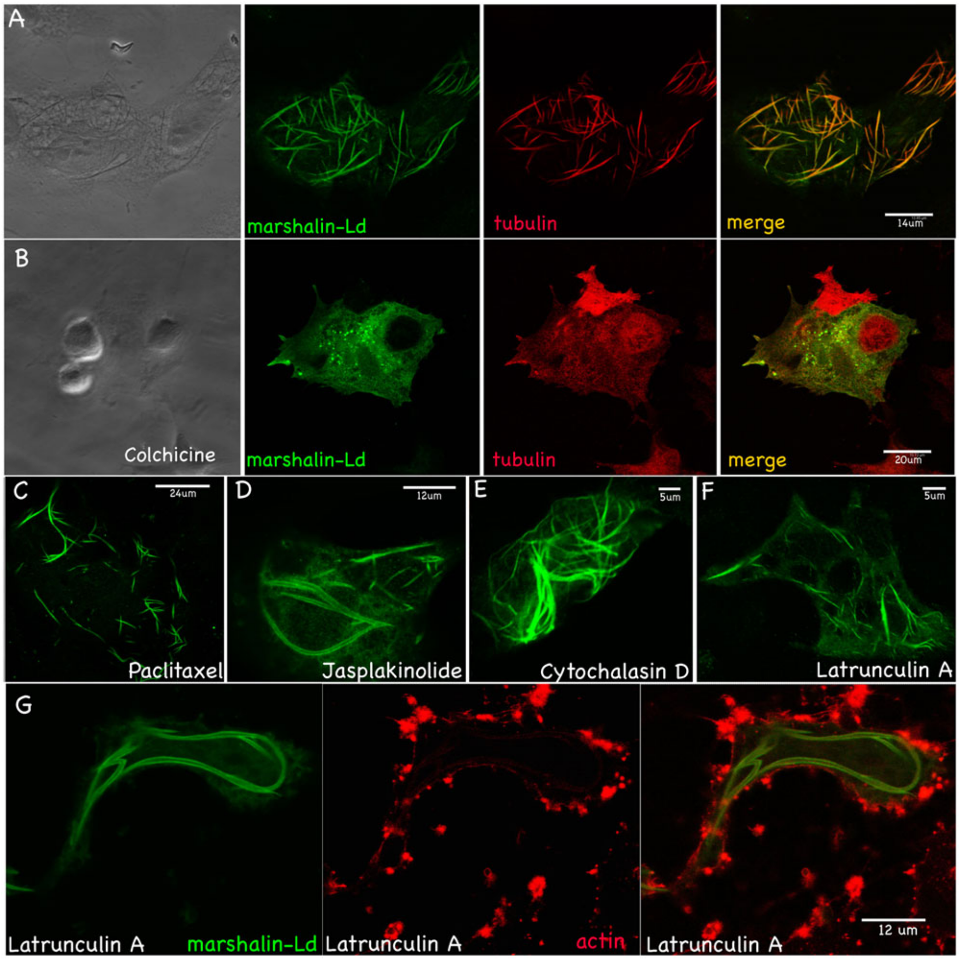

Fig. S4. MT bundles induced by *marshalin-Ld* cDNA. Marshalin-Ld-V5-expressing OK cells stained with anti-V5 for detecting marshalin (green) in panels A–G, anti-α-tubulin for detecting MTs (red) in panels A-F, and Texas Red-X phalloidin for actin filaments in panel G. All images acquired 48 hrs post transfection. (A) Cells without drug treatment. (B) Cells treated with 250 ng/μl colchicine, a microtubule polymerization inhibitor, for 24 hrs. (C) Cells treated with 0.5 μM paclitaxel, a microtubule stabilizer, for 3 hrs. (D) Cells treated with 10 nM jasplakinolide, which increases actin filament assembly, for 24 hrs. (E) Cells treated with 0.5 μM cytochalasin D, which inhibits actin polymerization, for 24 hrs. (F) Cells treated with 0.25 μM latruculin A for 24 hrs, which also inhibits actin polymerization as shown in panel G. Scale bars: 14 μm (A), 20 μm (B), 24 μm (C), 12 μm (D,G), 5 μm (E,F).

Table S1. Marshalin isoforms and their accessory numbers.

Name	Exons	Amino acids	Accession number
Marshalin-La (v1)	19 exons: Exon1-19	1280 aa	KC426930
Marshalin-La (v2)	19 exons: Exon1-19	1279 aa	KC426931
Marshalin-Lb (v1)	18 exons: Exon1-4, E6-19	1269 aa	KC426932
Marshalin-Lb (v2)	18 exons: Exon1-4, E6-19	1268 aa	KC426933
Marshalin-Lc (v1)	18 exons: Exon1-5, E7-19	1264 aa	KC426934
Marshalin-Lc (v2)	17 exons: Exon1-5, E7-19	1263 aa	KC426935
Marshalin-Ld (v1)	17 exons: Exon1-4, E7-19	1253 aa	KC426936, NM_001163749.1
Marshalin-Ld (v2)	17 exons: Exon1-4, E7-19	1252 aa	KC426937, NM_027171.3
Marshalin-Sa (v1)	19 exons: Exon1-19 (partial Exon13)	864 aa	KC426938
Marshalin-Sa (v2)	19 exons: Exon1-19 (partial Exon13)	863 aa	KC426939
Marshalin-Sb (v1)	18 exons: Exon1-4, E6-19 (partial Exon13)	853 aa	KC426940
Marshalin-Sb (v2)	18 exons: Exon1-4, E6-19 (partial Exon13)	852 aa	KC426941
Marshalin-Sc (v1)	18 exons: Exon1-5, E7-19 (partial Exon13)	848 aa	KC426942
Marshalin-Sc (v2)	17 exons: Exon1-5, E7-19 (partial Exon13)	847 aa	KC426943
Marshalin-Sd (v1)	17 exons: Exon1-4, E7-19 (partial Exon13)	837 aa	KC426944
Marshalin-Sd (v2)	17 exons: Exon1-4, E7-19 (partial Exon13)	836 aa	KC426945

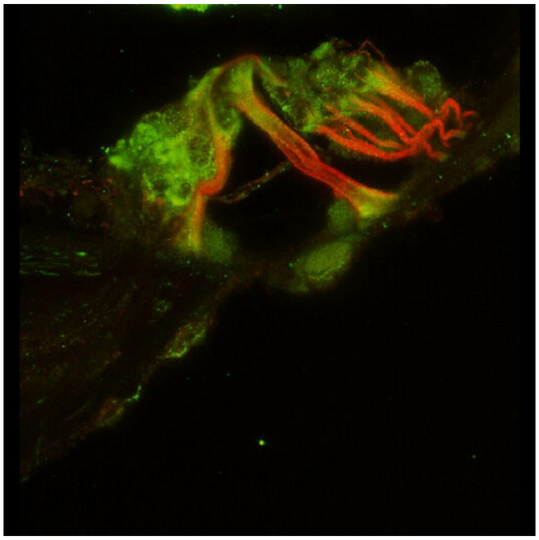

Movie 1. 3D-movie showing the location of marshalin in the organ of Corti. Thirty individual images were collected from a P27 mouse cochlea at intervals of 0.15 µm per section. The movie was created using ImageJ.

Biology Open
